# Consumption of Cisatracurium in different age groups, using a closed loop computer controlled system

**DOI:** 10.1186/1471-2253-14-29

**Published:** 2014-04-21

**Authors:** Shehzaad Joomye, Donglai Yan, Haiyun Wang, Guoqiang Zhou, Guolin Wang

**Affiliations:** 1 Department of Anaesthesiology, Tianjin Medical University General Hospital, Tianjin Research Institute of Anaesthesiology, No. 154 Anshan Road, Heping District, 300052 Tianjin, China; 2 Department of Anaesthesiology, Tianjin Second People’s Hospital, NO.7 South Road, Nankai District, 300192 Tianjin, China

**Keywords:** Age, Anesthesia, Cisatracurium, Closed-loop computer controlled infusion system, Recovery index, Sufentanil, Propofol

## Abstract

**Background:**

We devised this study to quantify the effect of age on the consumption of cisatracurium under general anaesthesia, using a computer controlled closed loop infusion system. We further investigated this effect on, sufentanil and propofol consumption.

**Methods:**

74 patients of physical status I and II, requiring general anaesthesia for elective abdominal surgery, were assigned to three groups. Patients in group 1 were aged from 20 to 45, group 2 were from 46 to 64, and group 3 above 65 years old. General Anesthesia was maintained with propofol and muscle paralysis was maintained using a closed-loop computer controlled infusion of cisatracurium. For analgesia, intermittent bolus of sufentanil 10 μg was given.

**Results:**

Cisatracurium consumption in group 1, 2 and 3 were 1.8 ± 0.3, 1.6 ± 0.4 and 1.3 ± 0.4 μg/kg/min respectively. There was significant difference of cisatracurium consumption between group 1 and 3 (P = 0.002), and the consumption of cisatracurium in group 3 was less as compared with group 2 (P = 0.04). The average recovery index of patients in group 1, 2 and 3 were 8.8 ± 2.6, 11.5 ± 2.9 and 12.7 ± 2.5 minutes respectively. There were difference between group 1 and 2 (P = 0.02). As compared with group 1, the recovery index was still longer in group 3 (P = 0.001). Patients in group 1, 2 and 3 consumed an average sufentanil 0.4 ± 0.1, 0.4 ± 0.1 and 0.3 ± 0.1 μg/kg/hr, respectively. There were statistical significant between group 1 and 3 (P < 0.0001), and the same trend was found between group 2 and 3 (P = 0.03). The Consumption of propofol in group 1, 2 and 3 were 5.1 ± 0.4, 4.3 ± 0.6 and 3.1 ± 0.5 mg/kg/hr. The difference in the propofol consumption was found statistically significant when comparing between any two groups.

**Conclusion:**

We concluded that the sensitivity of anesthetic agents increased with age. Less medication was required to achieve a desirable effect in older patients specially those above 65 years of age, and the drug effect was prolonged.

**Trial registration:**

ClinicalTrials.gov Identifier: NCT01785446.

## Background

Aging is a universal and progressive physiologic process characterized by decline end-organ reserve, decrease functional capacity, increasing imbalance of homeostatic mechanisms, and an increasing incidence of pathologic process [1]. With the consideration on the daily increase of surgery on elderly patients, proper adjustment of drug dosage is required for safe recovery from general anesthesia. To achieve this goal, anesthesiologists should minimize the dosage of anesthetics to avoid the side effect of drug residual, while maintains adequate intra-operative unconsciousness, analgesia and paralysis for older patients. Over-dosage of neuromuscular blocking agents especially in elderly patients can lead to delay in recovery and post-operative residual curarization [2]. Accurate administration of such drugs minimizes such effect. The closed-loop infusion is a very sensitive technique in providing stable surgical operating conditions, while diffusing minimal amount of drug to maintain relaxation at a given level [3]. Such technique allowed us not only to calculate accurately its consumption but also to prevent side effects due from over dosage. In this study, we determined the intra-operative consumption of cisatracurium infusion and its recovery index using a closed loop computer controlled system in different aged groups. We also calculated sufentanil and propofol consumption in these groups. The implication of this study may be reassuring to the clinician the effect of age on the profile of muscle relaxant, thus minimize the over dosage and prolonged recovery time.

## Methods

The study was approved by our hospital ethical committee: Board Name; Tianjin Medical University General Hospital Ethics Committee, Board Affiliation; Tianjin Medical University General Hospital Ethics Committee. (Phone: +86-22-60361517). Approval number: 201252.

It was conducted according to the guidelines of the above mentioned ethical requirements, and all patients gave written consent prior the study. Experimental Design and Groups Patients scheduled for elective abdominal general surgery under general anaesthesia were assigned to 3 groups. All patients were of ASA status I and II, and they were all required to give consent before starting the study. Group 1 were aged from 20 to 45, group 2 from 46 to 64 and group 3 were 65 and above. The exclusion criteria were as follows: patients with neuromuscular junction disorders, myopathies, peripheral neuropathies, encephalopathies, abnormal renal or liver laboratory function test and any patients with flaccid paralysis [4-6] (Additional file 1).

For accurate comparison of drug consumption between groups, all patients must have same induction protocol: midazolam 0.05 mg/kg, sufentanil 0.3 μg/kg, etomidate 0.2 mg/kg and a bolus dose of cisatracurium 0.15 mg/kg which was given by the computerized closed loop infusion system (CLMSRI-I, Guangxi VERYARK Technology Co., Ltd.). All patients received propofol infusion titrated to the clinical situation in a range of 4.5 to 7.5 mg/kg/hr (75–125 μg/kg/min) according to the BIS value of 40–60. Hypotension, defined as systolic blood pressure below 80 mmHg or mean arterial pressure below 60 mmHg for more than 5 minutes, was treated by reducing propofol infusion by 0.6 mg/kg/hr (10 μg/kg/min), but within the range of 4.5 to 7.5 mg/kg/hr. Additional intravenous fluids were given as deemed appropriate. Response was reassessed at 5 minute intervals and the above measures continued until stabilization of blood pressure. Hypertension, defined as systolic blood pressure above 150 mmHg or mean arterial pressure above 95 mmHg for more than 5 minutes, was treated by giving additional sufentanil (10 μg) boluses. Sufentanil boluses (10 μg) were also given to patients in all groups when there was an increase in the heart rate by more than 20 beats per minute or mean arterial pressure by more than 15% indicating lightening of anaesthesia. Response was reassessed at 5 minute intervals and the above measures repeated until stabilization.

During the whole procedure of anesthesia, the infusion of cisatracurium and the monitoring of neuromuscular block status were done by the computerized closed loop infusion system (CLMSRI-I, Guangxi VERYARK Technology Co., Ltd.). The degree of neuromuscular blockade was assessed every 20 seconds. Surface electrodes were attached over the ulnar nerve and over the first interosseus muscle and the index finger [7]. The train-of-four (TOF) sequence was used (2 Hz frequency, 100 ms pulse width), the stimulus output being a rectangular wave with a current of 0–70 mA. The machine calibrated automatically by searching for the optimal signal levels before setting the supramaximal level. Control electromyographic values were obtained after induction and following this, a stable baseline calibration signal was awaited and a second calibration was performed approximately 10 minutes after induction of anesthesia. During this period patients were ventilated manually with a mask. The degree of neuromuscular blockade was defined as the ratio of the measurement of the first twitch in the TOF sequence to the corresponding control value. The desired level of neuromuscular block (i.e., the set point) was set to 90% (the first twitch (T1) in the TOF sequence = 10% from control). Patients were intubated when the T1% dropped below 10% as compared with baseline value.

After obtaining a stable calibration signal, a bolus dose of cisatracurium 0.15 mg/kg was administered. We used the ideal body weight (IBW), as defined by Devine’s equation, for the calculation of the dose of cisatracurium [8]. Tracheal intubation was performed and the patients were mechanically ventilated using either of the above mentioned gas mixtures. Bolus administration of cisatracurium was followed by infusion of cisatracurium by a model-driven closed-loop feedback system as described previously [9]. The infusion rate maintained itself at the low rate of 0.2 μg/kg/min as long as the T1 response was less than 10%, which would automatically increase to 5.0 μg/kg/min when T1 is above 10%. The rate of infusion would fall back to 0.20 μg/kg/min when the T1 is less than 10%. Once the bolus dose was given after which the infusion rate was set at 0.20 μg/kg/min. Patients were intubated when the T1% dropped below 10%. The degree of neuromuscular blockage was assessed every 20 seconds throughout the procedure using the TOF testing. The consumption of cisatracurium was recorded in μg/kg/hr.

Propofol infusion was used in all patients for the maintenance of anesthesia intraoperatively. The rate of infusion was adjusted according to the BIS value (40–60). The total amount of propofol used from induction to skin closure was recorded and its consumption was calculated in mg/kg/hr in each patient. Sufentanil consumption was calculated in μg/kg/hr. The infusion of cisatracurium and propofol were stopped at skin closure. Furthermore, the linear regression analysis was used to establish the relationship between cisatracurium consumption and age.

After which the TOF monitoring was observed, and reversal agents, neostigmine 40 μg/kg with atropine 0.15 mg/kg [10], were given only when at least two twitches were present according to TOF monitoring. The tracheal extubation was performed after full recovery from neuromuscular block (TOF-ratio ≥ 0.90) [10], the monitoring of airway patency, respiratory rate, continuous oxygen saturation, blood pressure, and pulse were controlled in emergence and recovery. Assessment of the neuromuscular function was based on both physical examination and the TOF monitor. The recovery index (RI), defined as the time required for T1 of the TOF to recovery from 25% to 75% of baseline [11]. Patients would not leave the operating room with the TOF ratio of less than 90%. Fully awake patients, with stable vitals and ability to move all limbs were then transferred to the Post-Anesthetic Care Unit (PACU) for further monitoring. Patients were observed for at least 60 minutes in the PACU before sending back to the ward. Patients should fulfill well defined criteria before being sent back to wards, including [12]: fully conscious oriented in time, space and person; able to maintain a clear airway and exhibit effective protective reflexes; able to lift head and hold up for more than 30 seconds; bilateral firm hand grips and movement of all extremities; clear vision without nystagmus; respiration and oxygenation returned to preoperative base level; stable cardiovascular function on acceptable level with no unexplained irregularity or uncontrolled bleeding; pain and emesis properly controlled and analgesic or antiemetic regime prescribed.

### Statistical analysis

The calculation of the number needed to find a difference of at least 20% (group 1 or 2 vs group 3; the mean cisatracurium consumption of group 1 and 2 are larger than 1.6 μg/kg/min, and standard deviation ≈ 0.3) with an alpha risk of 5% and a power (1-beta) of 80% shows that 20 patients are needed in each group. The SPSS Statistics17.0 software was used for the statistical analysis. All results are given as mean ± SD. Test of normality was done for each group and one-way ANOVA was used to compare means between the 3 groups followed by Turkey test.

## Results

Table 1 summarizes the demographic data and results of the three groups. There was no significant difference in gender, body mass index and BIS value within the groups.

**Table 1:** The demographic data and results of the three groups

	**Group 1(age 20–45)**	**Group 2(age 46–65)**	**Group 3(age >65)**
Number of patients	21	33	20
Age	38.0 ± 7.0	54.7 ± 4.5	73.6 ± 3.2
Weight	67.1 ± 8.6	67.6 ± 10.5	61.2 ± 14.4
BMI	24.3 ± 2.16	24.7 ± 3.2	21.8 ± 1.8
Male/Female	7/14	12/21	6/14
Cisatracurium consumption, μg/kg/min	1.8 ± 0.3	1.6 ± 0.4	1.3 ± 0.4
Recovery index, minutes	8.8 ± 2.6	11.5 ± 2.9	12.7 ± 2.5
Sufentanil consumption, μg/kg/hr	0.4 ± 0.1	0.4 ± 0.1	0.3 ± 0.1
Propofol consumption, mg/kg/hr	5.1 ± 0.4	4.3 ± 0.6	3.1 ± 0.5

**Figure 1:**
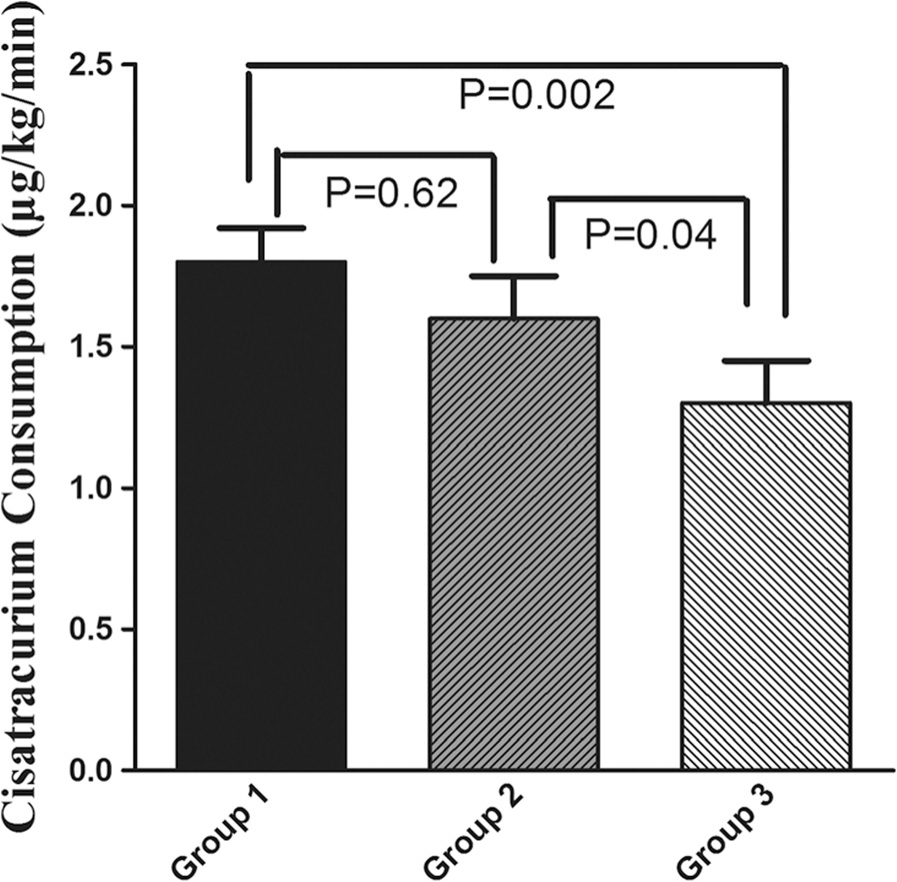
**The changes in cisatracurium consumption in the three groups.** Group 1: patients aged from 20–45; group 2: aged from 46–65; group 3: aged exceed 65. All results are given as mean ± SD.

**Figure 2:**
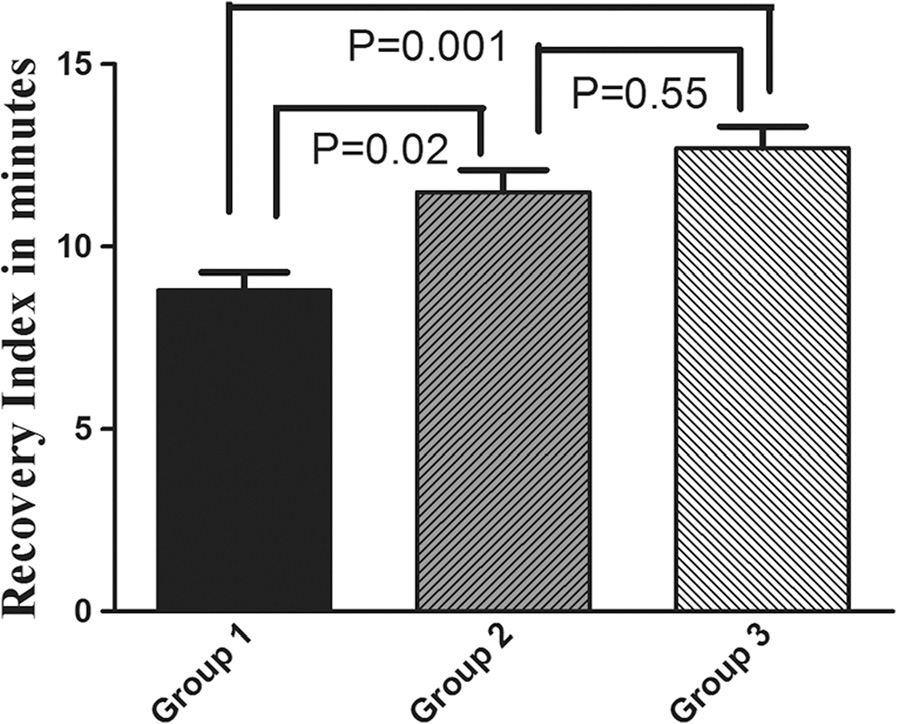
**The recovery index of the three groups.** Group 1: patients aged from 20–45; group 2: aged from 46–65; group 3: aged exceed 65. All results are given as mean ± SD.

**Figure 3:**
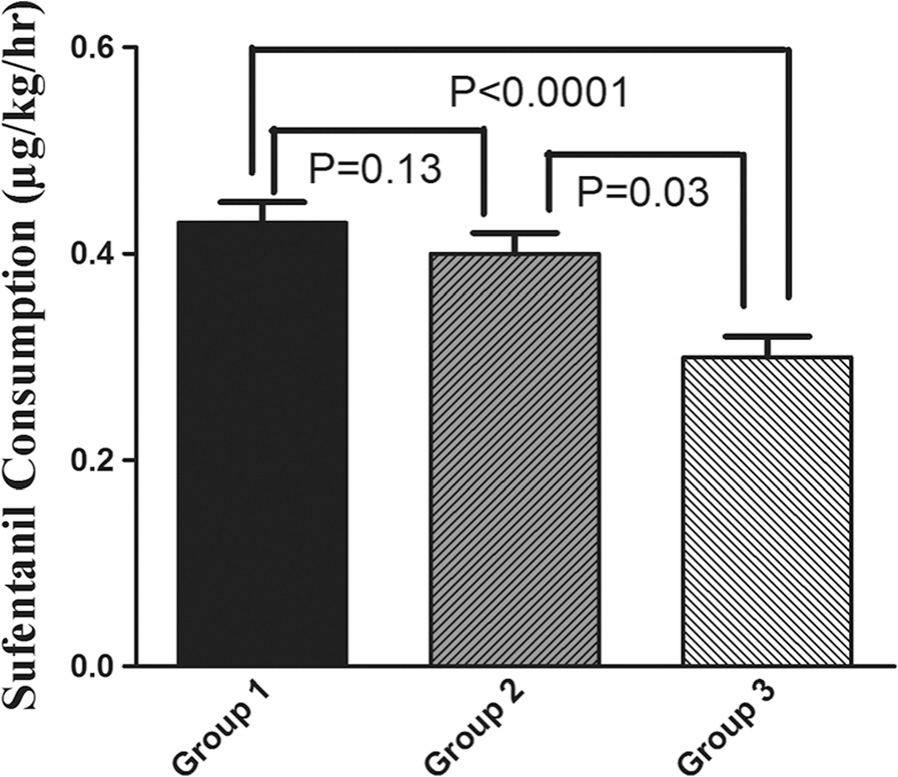
**Sufentanil consumption in the three groups.** Group 1: patients aged from 20–45; group 2: aged from 46–65; group 3: aged exceed 65. All results are given as mean ± SD.

**Figure 4:**
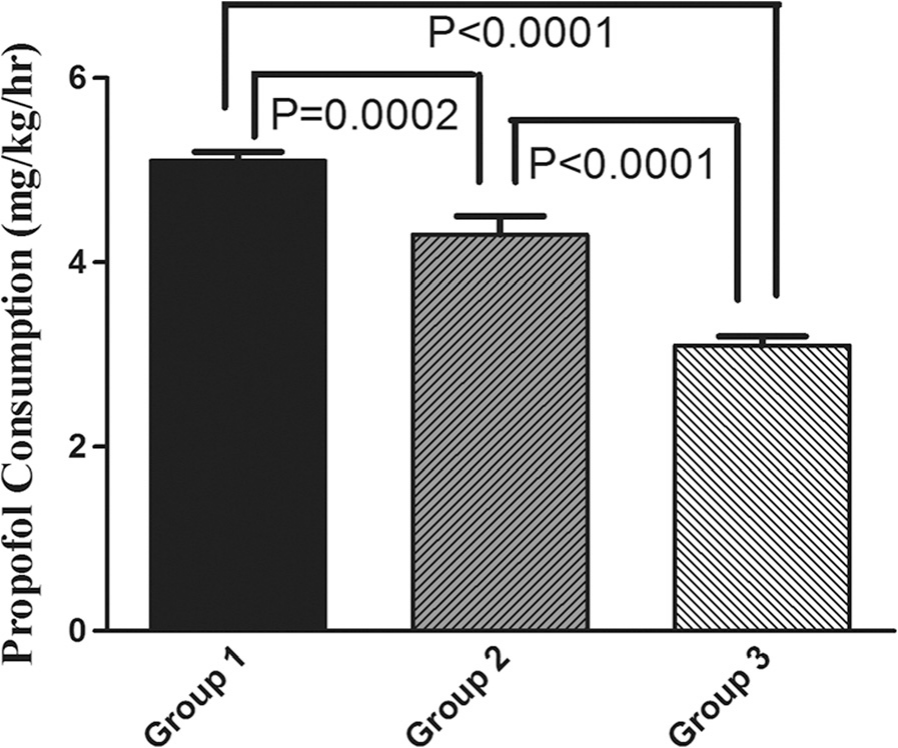
**Propofol consumption in the three groups.** Group 1: patients aged from 20–45; group 2: aged from 46–65; group 3: aged exceed 65. All results are given as mean ± SD.

The average consumption of cisatracurium in group 1, 2 and 3 were 1.8 ± 0.3, 1.6 ± 0.4 and 1.3 ± 0.4 μg/kg/min respectively. Figure 1 demonstrates a box plot showing the decreasing consumption of cisatracurium in the three groups. There was significant difference of cisatracurium consumption between group 1 and 3 (P = 0.002), and the consumption of cisatracurium in group 3 was less as compared with group 2 (P = 0.04). The increase in the sensitivity of cisatracurium with age was further demonstrated by its increase in recovery index. The average recovery index of patients in group 1, 2 and 3 were 8.8 ± 2.6, 11.5 ± 2.9 and 12.7 ± 2.5 minutes respectively (Figure 2). There were difference between group 1 and 2 (P = 0.02). As compared with group 1, the recovery index was still longer in group 3 (P = 0.001). We also found a decrease in consumption of sufentanil with age (Figure 3). Patients in group 1, 2 and 3 consumed an average sufentanil 0.4 ± 0.1, 0.4 ± 0.1 and 0.3 ± 0.1 μg/kg/hr, respectively. There were statistical significant between group 1 and 3 (P < 0.0001), and the same trend was found between group 2 and 3 (P = 0.03). Figure 4 illustrates the change in propofol consumption. The Consumption of propofol in group 1, 2 and 3 were 5.1 ± 0.4, 4.3 ± 0.6 and 3.1 ± 0.5 mg/kg/hr. The difference in the propofol consumption was found statistically significant when comparing between any two groups.

To further confirm the relationship between cisatracurium consumption and age, we performed a linear regression analysis. The linear regression model showed an inverse and significant relationship between cisatracurium consumption and age (p <0.01).

For the unequal number of patients in three groups, we randomly selected 20 patients from group 2 and reanalyzed the statistical results, all these procedures repeated for three times. We still found the same results as compared with the previous one.

## Discussion

This study was designed to better appreciate the impact of age in the consumption of cisatracurium with a closed loop computer controlled system. We also studied the effect of age on the recovery index, and the consumption of sufentanil and propofol. We noted a decrease in consumption of cisatracurium with age, and there was a decrease of 26% in group 3 (older) as compared with group 1 (young patient). A total number of 74 patients were investigated and were divided into 3 groups according to their age. We decided to have more than two groups to better appreciate the linear regression of consumption of drugs with age. The pharmacodynamics of muscle relaxants are altered in elderly patients since hepatic metabolism and renal clearance decreases with age. We would expect that the effect of cisatracurium would be unaffected with age since it is eliminated by Hofmann degradation. But our results showed an increase in the effect of cisatracurium with age. Besides the renal and hepatic physiologic changes with age, there are a number of other physiologic changes that accompany the aging process, including decrease in total body water, decrease in lean body mass and muscle mass, increase in total body fat, decrease in hepatic and renal blood flow, and decrease in cardiac reserve, which account for the altered responses to muscle relaxants [13,14] in aged patients. We might infer that a decrease in total body water could lead to a smaller central compartment and increased serum concentration after bolus dose administration of a drug. In addition the increase in body fat might result in greater volume distribution, with the potential to prolong the clinical effect of a given medication [15]. Furthermore it had been postulated by Cope TM and colleagues that the effect of mivacurium was enhanced in older patients due to a decrease of plasma acetyl cholinesterase activity [16]. Sorooshian SS et al., studies the pharmacodynamics and pharmacokinetics of cisatracurium in young and elderly adults. They found that the plasma clearance of cisatracurium was similar in young and the elderly group, but the volume of distribution was significantly less in the elderly which accounted for delayed response of cisatracurium [17].

Atracurium which also undergoes Hofmann degradation was also found by Kitts and colleagues [18] to have an increasing effect in elderly patients. They concluded that the pharmacokinetics, but not the pharmacodynamics, of atracurium differ significantly between elderly and young adults which resulted in a slightly prolonged effect of neuromuscular blockade in elderly patients. Kisor et al. [19] found that Hofmann clearance accounted for 77% of total body clearance. Organ clearance was 23% of total body clearance. Renal clearance, a component of organ clearance, was 16% of total body clearance, whereas renal clearance of atracurium accounted for 2% to 10% of the administered dose, values slightly lower than those found for cisatracurium [20]. Therefore, we think the deteriorating renal function with ages also contributed to the longer effect of cisatracurium in the older age groups. The average age in group 3 is 73.6 ± 3.2 yr, In a review of Cope et al., they stated that the physiological changes such as the decrease in cardiac output and muscle mass, an increase in body fat and the deterioration in renal and hepatic function, which could affect the pharmacokinetics of neuromuscular-blocking agents may not become apparent clinically in healthy individuals until the age of at least 75 years [16]. In our current study, the average age in group 3 is 73.6 ± 3.2 yr, which is close to the age they inferred.

We also demonstrated a decrease in consumption of sufentanil by 32% in patients above 65 years of age as compared to patients below 45. Similar results have also been obtained in other studies. Shafer provided a comprehensive review of the pharmacology of sufentanil, alfentanil, and fentanyl in elderly patients [15]. Sufentanil, alfentanil and fentanyl were approximately twice as potent in elderly patients. These findings were primarily related to an increase in drain sensitivity to opioids with age, rather than alterations in pharmacokinetics.

We further investigated the impact of patients’ age on propofol consumption. Patients below 45 years of age consumed an average of 5.1 ± 0.4 mg/kg/hr of propofol. Older patients between 46 and 64 consumed 4.3 ± 0.6 mg/kg/hr, and elderly patients above 65 consumed 3.1 ± 0.5 mg/kg/hr. This showed a decrease of 39% in consumption between group 1 and 3. For an explanation of the observed results, both pharmacokinetic and pharmacodynamic mechanisms must be considered, i.e. that a defined dose of propofol produces higher drug concentrations in an elderly patient and that the same propofol concentration produces a more profound anaesthetic effect [15]. When comparing the pharmacokinetics of a propofol bolus, Kirkpatrick and colleagues [21] could demonstrate that mean propofol blood concentrations tended to be higher in elderly (65–80 yr) than in younger (18–35 yr) patients, best explained by a significantly lower metabolic propofol clearance and a smaller volume of the central compartment. Furthermore, Schüttler and Ihmsen found a linear decrease of the propofol elimination clearance in patients older than 60 yr [22]. These findings may be explained by a reduction of liver function or liver blood flow in elderly patients [23], as the propofol clearance has been characterized as liver blood flow dependent [24]. For propofol pharmacodynamics, age-related changes of brain function must be considered. Comparing a 20- and a 90-yr-old individual, brain undergoes an age-dependent weight reduction of approximately 10%, while the gray matter decreases more than the white matter [25]. Concomitantly, the cell number of the frontal cortex decreases by approximately 40% [26]. Our clinically obtained data are supported by results of Schnider and colleagues [27] who, in a laboratory setting, reported that elderly patients were more sensitive to hypnotic and EEG effects of propofol than were younger persons.

## Conclusions

In conclusion, we summarized that the sensitivity of cisatracurium increases with age of patients. Less cisatracurium was required to achieve a desirable effect in elder population with a closed loop infusion system. Even though the dose decreases in the elder patients, a prolonged duration of action of cisatracurium was observed among patients above 65 years of age. We also found that the consumption of propofol and sufentanil decrease in elderly as compared with young adult and middle adult population. These implied for the clinicians that older patients would need a downward adjustment in medication dose during general anesthesia.

## Competing interests

The authors declare that they have no competing interests.

## Authors’ contributions

SJ helped design the study, conduct the study, analyze the data, and write an initial draft of manuscript. DY recruited the patients, performed many of measurements, data analysis, and manuscript revisions. HW contributed to study design, patient recruitment, data analysis, and manuscript preparation. GZ contributed to patient recruitment, data collection and analysis, and manuscript preparation. GWdesigned the study, collected and analyzed the data, and revised the manuscript. All authors read and approved the final manuscript.

## Authors’ information

Donglai Yan is co-first author.

## Pre-publication history

The pre-publication history for this paper can be accessed here:

http://www.biomedcentral.com/1471-2253/14/29/prepub

## Supplementary Material

Additional file 1 CONSORT 2010 Flow Diagram. Click here for file
