# Supplementary material for: Consumption of Cisatracurium in different age groups, using a closed loop computer controlled system
Source: BMC Anesthesiol. 2014 Apr 21;14:29. doi: 10.1186/1471-2253-14-29 (PMC4021420; doi:10.1186/1471-2253-14-29)
Supplement: Additional file 1 — CONSORT 2010 Flow Diagram. [file 1471-2253-14-29-S1.doc]

**CONSORT 2010 Flow Diagram**

**Allocation**

**Analysis**

**Follow-Up**

**Enrollment**

Assessed for eligibility (n=80 )

Excluded (n= 6 )

♦  Not meeting inclusion criteria (n= 0 )

♦  Declined to participate (n= 4 )

♦  Other reasons (n= 2 )

Analysed (n=21 )
♦ Excluded from analysis (give reasons) (n= 0 )

Lost to follow-up (give reasons) (n=0 )

Discontinued intervention (give reasons) (n=0 )

Allocated to intervention (n=21 )

♦ Received allocated intervention (n= 21 )

♦ Did not receive allocated intervention (give reasons) (n=0 )

Randomized (n=74 )

Lost to follow-up (give reasons) (n=0 )

Discontinued intervention (give reasons) (n=0 )

Analysed (n=33 )
♦ Excluded from analysis (give reasons) (n= 0 )

Analysed (n=20 )
♦ Excluded from analysis (give reasons) (n= 0 )

Allocated to intervention (n=20 )

♦ Received allocated intervention (n=20 )

♦ Did not receive allocated intervention (give reasons) (n= 0 )

Lost to follow-up (give reasons) (n=0 )

Discontinued intervention (give reasons) (n=0 )

Allocated to intervention (n=33 )

♦ Received allocated intervention (n=33 )

♦ Did not receive allocated intervention (give reasons) (n= 0 )
